# Mr. Bulley on Treacle (Molasses) and Water for Burns

**Published:** 1842-06-18

**Authors:** 


*Treacle (Molasses) and Water for Burns.*—Mr. Bulley used this with very good effect in a case of scald from melted pitch. In the first instance he employed a paste composed of equal parts of treacle and flour. We need not mention the particulars of the case, but content ourselves with the concluding remarks of Mr. Bulley.

"In recording the treatment adopted in the foregoing case, I do not wish to take any credit to myself for employing a remedy with the virtues of which, in such cases, surgeons have been long acquainted. Mr. Greenhow, of Newcastle, first introduced it into practice, using it as a defensative, for the purpose of preventing the access of air to the denuded parts. He did not, I believe, continue to use it throughout the whole progress of the case, but substituted for it other applications which the circumstances of the case might afterwards seem to require. In the commencement of the preceding case I used it, mixed with flour, for the same purpose of excluding air, but, finding it occasioned pain, and that I could not properly see what was going on underneath, although it had seemed to promote the growth of granulation, I determined to use equal parts of treacle and water, on rags, constantly applied as a lotion to the injured surface. The application of treacle in this manner has convinced me by the result of this, and other cases similarly treated, that it has some specific effect in expediting the cicatrisation of burns and scalds, however extensive they may be, and that it prevents, in a great degree, the unsightly puckering and contraction which too often interfere with the proper actions of joints involved in these accidents. I have, since that time, had several opportunities of testing its value as a remedy in these cases; and have, from what I have seen of its effects, adopted it in every case of the kind which has come under my care, both in hospital and private practice; and in each case it has seemed to have been instrumental in preventing, or at least diminishing, the chances of consecutive contraction."—*Provincial Med. and Surg. Journ.* Feb. 5, 1842.

